# Protein Surface Characterization Using an Invariant Descriptor

**DOI:** 10.1155/2011/918978

**Published:** 2011-11-22

**Authors:** Zainab Abu Deeb, Donald A. Adjeroh, Bing-Hua Jiang

**Affiliations:** ^1^Lane Department of Computer Science and Electrical Engineering, West Virginia University, Morgantown, WV 26506, USA; ^2^Department of Pathology, Anatomy and Cell Biology Jefferson Medical College, Thomas Jefferson University, Philadelphia, PA 19107, USA

## Abstract

*Aim*. To develop a new invariant descriptor for the characterization of protein surfaces, suitable for various analysis tasks, such as protein functional classification, and search and retrieval of protein surfaces over a large database. *Methods*. We start with a local descriptor of selected circular patches on the protein surface. The descriptor records the distance distribution between the central residue and the residues within the patch, keeping track of the number of particular pairwise residue cooccurrences in the patch. A global descriptor for the entire protein surface is then constructed by combining information from the local descriptors. Our method is novel in its focus on residue-specific distance distributions, and the use of residue-distance co-occurrences as the basis for the proposed protein surface descriptors. *Results*. Results are presented for protein classification and for retrieval for three protein families. For the three families, we obtained an area under the curve for precision and recall ranging from 0.6494 (without residue co-occurrences) to 0.6683 (with residue co-occurrences). Large-scale screening using two other protein families placed related family members at the top of the rank, with a number of uncharacterized proteins also retrieved. Comparative results with other proposed methods are included.

## 1. Introduction

The Protein Data Bank (http://www.pdb.org/pdb/home/home.do) (PDB) currently has more than 3000 protein structures classified as uncharacterized or as proteins of unknown function. This is about 5% of the total structures in PDB. The Pfam database was recently reported to contain over 2200 gene families with unknown function [1]. It has been argued that there are even more local regions on the protein structures that are not completely characterized, and whose functions are not known [2]. Therefore, with the increasing rate at which protein structures are being generated, the problem of protein function annotation has become a major challenge in the postgenomic era [3–5]. The function of a given protein is largely determined by its three-dimensional structure [6]. The specific shape and orientation of a protein in 3D space are key elements that determine how the protein interacts with its environment, and hence the function of the protein. Although related proteins often have similar functions, it is well known that sequence similarity between proteins does not always lead to functional similarity [7, 8]. Even different functions have been observed for structures with the same fold [9]. Conversely, sequences have been observed with low sequence similarity, but highly structural and functional similarity [10]. The trypsin-like catalytic triad [9] is one example of proteins with different folds, but similar functions. A similar argument can be made between sequence and surface, and between surface and fold. While residues on the protein surface typically make up a small percentage of the total residues in a protein, they often represent the most conserved functional elements of the protein [11]. Therefore, analyzing protein structures using information about their 3D surfaces is essential in the quest for protein function annotation, especially in the study of functional similarities between nonhomologous proteins. 

At the core of most activities in the analysis of protein structures and protein function is similarity measurement between structures. Such measurements must deal with different levels of structural similarity, arbitrary mutations, deletions, and insertion of residues, local surface similarities, and so forth. When the problem is similarity measurement between protein surfaces, a major issue becomes how the protein surface is represented, and how the representation can be used for the required similarity measurement. Another problem is that of computation. Structure alignment, the basis for most approaches to protein 3D structure analysis is known to be NP-hard [12]. A major difficulty in comparing protein surfaces locally is the problem of matching 3D structures, since structures need to undergo an exhaustive amount of rotation and translation in order to obtain an adequate structural alignment and to perform an accurate matching [8]. Clearly, a method that avoids the step of local structural alignments can have a significant advantage, especially in screening of similar surfaces over a large database. 

In this paper, we introduce an invariant descriptor for the characterization of protein surfaces. We then use this characterization to study the problem of classifying proteins into their functional families based primarily on their surface characteristics. This is a challenging problem, but one that is important in the quest for functional annotation of proteins, using information from potentially nonhomologous proteins. We also show how we can use such a descriptor in various related analysis activities, such as in effective retrieval of similar protein surfaces from very large databases, such as the Protein Data Bank (PDB).

## 2. Background and Related Work

### 2.1. Protein Sequences, Structure, and Surface

**Figure 1:**
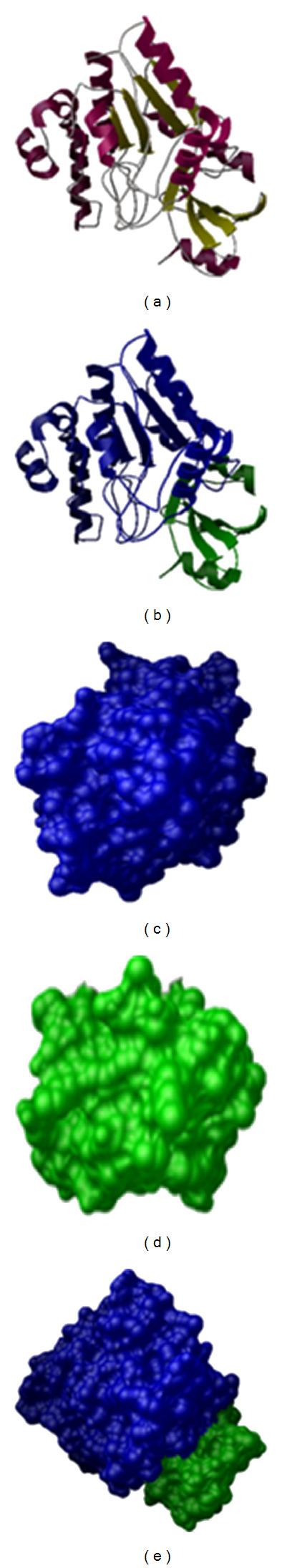
Protein structures for a sample protein (PDB id: 2UDI). (a) Secondary structure elements—*α*-helixes (magenta), *β*-sheets (gold), and turns (gray); (b) two chains: chain E (blue), chain I (green); (c) surface and 3D shape for chain E; (d) surface and 3D shape for chain I; (e) quaternary structure for the protein. Figures are produced using PMV [16].

Although proteins could vary significantly in their functions and 3D shapes, they also share a general common structure. Proteins are composed of 20-amino acids that are connected via peptide bonds [13]. Each protein is composed of an ordered sequence of amino acids. The order in which these amino acids are connected is called the *protein sequence*, or the *primary structure* of the protein [14]. This primary sequence determines the 3D structure of the protein. All proteins are composed of four common structural types: primary structure, secondary structure, tertiary structure, and quaternary structure. The primary structure is simply the amino acid sequence. The *secondary structure* is formed by patterns of intermolecular bonding of hydrogen and is determined primarily by the location and the directions of these patterns [14, 15]. This is often described in terms of secondary structural elements (SSEs), such as *α*-helixes, *β*-sheets, and turns. The overall 3D shape of the secondary structures determines the *tertiary structure* of the protein. When two or more chains combine to form a larger molecule, the whole structure is called the *quaternary structure*.  Figure 1 shows an example of some of the common protein structural types (the sequence is not included). 

A common method for protein function prediction is by annotation transfer from known homologous proteins [17]. Functions of novel proteins can be determined by sequence comparisons, for instance using sequence alignment. When proteins evolve, the protein structure remains more highly conserved when compared to the sequence. Protein sequences change more easily during evolution due to residue mutations, for instance by substitution, insertion, or deletion. Hence, proteins that belong to the same family (homologous proteins) may not be identified using sequences alone. Orengo et al. [17] reported that proteins related to the same family could share fewer than 15% identical residues. The protein structure retains a significant portion of similarity even between distant homologs. In general, the degree of structural or sequence similarity varies substantially between protein families. Some families can handle more changes than others. This so-called *structural plasticity* [17] has a considerable impact on the functionality of some proteins, or members of a protein family. A consideration of the protein structure and its variability becomes important in such situations for further analysis of functional similarity between proteins.

A classical approach for deriving the protein function is by first determining its 3D structure, which can then provide some ideas about its function [17]. Protein 3D structures provide information about the binding sites, active sites, and how proteins interact with each other, and thus could provide an insight into the function of the protein [17]. How proteins interact with each other and with other molecules (e.g., ligands) is determined primarily by the amino acids on the protein surface [18]. Therefore, knowledge of the protein surface residues could help in a better understanding of what molecules are binding together, and in some cases, why they bind [18]. The protein surface could also provide significant information about protein functions which cannot be easily detected, even in the presence of sequence or fold similarity. Therefore, the analysis of protein surfaces is important in the study of intermolecular interactions. Clearly, advances in our understanding of protein surfaces could have important implications in various biomedical fields, such as personalized medicine, drug discovery, drug design, and so forth.

### 2.2. Protein Surface Characterization Methods

Given the foregoing, it is not surprising that different methods have been proposed to characterize the protein surface. Popular examples include those based on surface shape distributions [19], Gauss integral [20], Fourier transform [21], spherical harmonics [22, 23], alpha-shapes [2, 24], and Zernike polynomials [7]. Contact maps between protein surfaces were studied in [25], while similarity networks between surface patches from protein binding sites were studied in [26, 27]. Protein surface similarity using varying resolutions of structural data have also been studied, for instance, using medium-resolution Cryo-EM maps in [26] and low resolution protein structure data in [28]. SHARP [29] provides a mechanism to predict protein-protein interaction by analyzing overlapping protein 3D surface regions. SURFACE [5] is a database of protein surface regions that can be useful for annotation.

Much earlier, Jones and Thornton [30] analyzed protein-protein interaction by using surface patches, where patches are defined based on the C_*α*_ atoms that have a predetermined accessible surface area, and adhere to defined constraints on the solvent vectors. Each patch is then described using six parameters, namely, solvation potential, residue interface propensity, hydrophobicity, planarity, protrusion, and accessible surface area. Ferrè et al. [5] analyzed locally similar structures by matching surface patches composed of subsets of amino acids. Each residue on the protein surface is represented using a vector joining its C_*α*_ atom and the centroid of its side chain atoms. Surface patches are then compared for similarity by comparing the residue vectors for all possible pairs of residues from the query and target surface patch. Matches are determined based on the root mean square distance, and the residue similarity as determined using a standard substitution matrix. The results of using this method on a nonredundant list of protein chains as recorded in the SURFACE database [5], a collection of protein surface regions that can be useful for annotation. Below, we describe three approaches that are more closely related to our work. See [28, 31] for reviews on surface comparison methods.

**Distance Distributions**

Distances, geometry, and topology have for long been used in the analysis of general protein 3D structures [32]. Residue distances have been used in standard texture-based analysis of 2D textures (distance matrices) formed by the distances between residues in a protein structure [33]. The use of topological invariants, as captured using Gaus integrals for the automated analysis and representation of general protein 3D structures was described in [20]. Much earlier, Connolly [19] proposed the analysis of protein surfaces using the notion of surface shape distributions. Essentially, surface shapes correspond to different geometric configurations defined on the protein surface. Binkowski and Joachimiak [11] proposed the use of surface shape signatures (SSSs) as a method to describe protein surfaces by exploiting global shape and geometrical properties of the surfaces. Shape signatures are computed based on the distances measured between each unique atom pairs on the surface. Distances are then sorted based on which their distributions are generated. With the distributions, the problem of matching between two surfaces is now reduced to that of comparing their distributions. Comparison between two distributions is performed using the Kolmogorov-Smirnov (KS) test. The use of the shape distribution is fast and relatively resilient to scale, rotation, and mirroring. However, the discrimination ability is still a problem, as the SSS tends to lose important surface details.

**Zernike Polynomials**

Following earlier work by Canterakis [34] on the use of 3D Zernikes for the analysis of general 3D objects, Sael et al. [7] introduced 3D Zernike to the area of protein structural similarity matching. Here, the protein 3D structure is represented as a series expansion of 3D Zernike functions. The triangulated Connolly surface of the protein is computed, and subsequently the protein is placed into a 3D cubic grid and voxelized. Each voxel has a value of **1** or **0**, depending on whether the voxel is on the protein surface or in the interior. The 3D Zernike function is then applied to the voxelized 3D protein shape to obtain the 3D Zernike descriptors. Therefore, the problem of comparison of 3D surfaces is reduced to that of comparing two vectors representing the 3D Zernike descriptors for each protein surface. Several distance measures were tried, such as the Euclidean distance, Manhattan distance, and a correlation-based distance defined as the complement of the correlation coefficient between two Zernike descriptors. Venkatraman et al. [23] studied the use of both spherical harmonics and 3D Zernike descriptors in the retrieval of functionally similar proteins. In a more recent work, Sael and Kihara [28] used the Zernike descriptor to study protein surfaces in low resolution data. Computation of the required Zernike polynomials is, however, known to be a major computational huddle [35]. This problem is even worse for the 3D Zernike polynomials needed for protein surfaces. Thus, the required preprocessing before matching is performed may be a problem for indexing and real-time search of large-scale datasets.

**Fingerprints**

A recent work [8] used the idea of extracting invariant fingerprints from patches on the protein surface. Patches are obtained by generating the dot surface of the protein and constructing a graph to approximate the protein surface. Afterwards, circular patches are generated as a contiguous surface area from a center point, where the radius of the patch is within a predetermined cutoff. Patches are created for each single point on the surface, after which a fingerprint representation of the patch is computed as a geodesic distance-dependent distribution of directional curvature. Geodesic distances are computed from the central vertex in each patch. Comparisons between fingerprints were performed using the average fingerprint similarity score (AFSS) and the direct fingerprint similarity score (DFSS). Final scores are computed after an alignment procedure based on the AFSS. Clearly, computational complexity will be a major problem here, especially given the computation of the patch representation for each vertex on the surface graph (number of vertices is much more than the number of surface residues). The need for a later stage of alignment for the final computation of matching scores only compounds the computational burden (see [12], for example).

The key difference in our method is the use of the local patch descriptors as defined by the distribution of distances between C_*α*_ atoms within each surface patch, conditioned on the specific residue at the center of the patch, and the particular residues found within the patch. Our method computes the residue-specific distance distributions, and residue-distance cooccurrences for the protein surface patches using only the C_*α*_ atoms on the protein surface. Residues in the interior of the protein are discarded. Unlike the approach in [8], we avoid the time complexity of generating a graph representation of the surface before the surface can be scanned to generate the patches and then compute the distance distribution. Further, ours does not depend on the time-consuming process of initial surface alignment.

## 3. Methods

We present an invariant descriptor for characterizing protein surfaces. We start with a local descriptor of selected circular patches on the protein surface. For a given surface patch, the local descriptor is computed based on the residue distances from the center of the patch. The descriptor records the distance distribution between the central residue and the residues within the patch, keeping track of the number of particular pairwise residue cooccurrences in the patch. A global descriptor for the entire protein surface is then constructed from the local descriptors by combining information from local descriptors with similar central residues. The proposed descriptor is invariant to rotations of the surface and mirroring. 

Using a fixed patch size, we obtain a descriptor for the protein surface, independent of the size of the protein structure. Thus, the descriptor can facilitate the rapid matching of protein chains, and will eliminate the need for the exhaustive alignment of the protein 3D structures. For a given protein structure or protein chain from a database, such as the PDB, the proposed method can be summarize in the following steps:

1. generate the Connolly surface [36] for the protein chain;
2. generate the surface patches and compute the local invariant descriptor for each patch on the surface;
3. compute the global invariant surface descriptor for the protein chain, by combining information from the local patch descriptors;
4. perform surface matching and comparison using the descriptors;
5. classify the protein into its potential functional family, or perform protein surface retrieval using the invariant descriptors.

**Figure 2:**
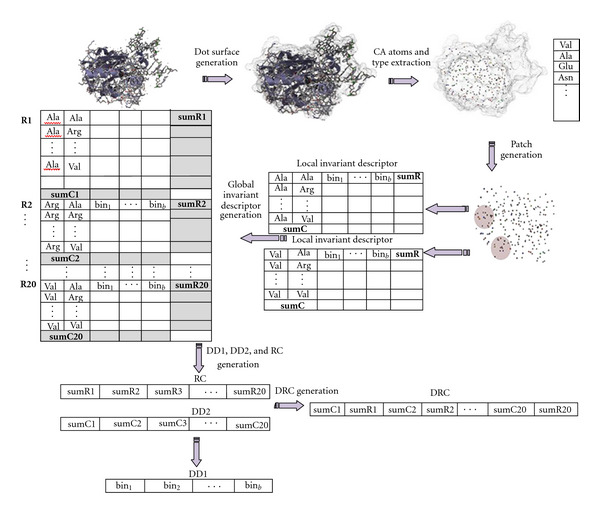
Schematic diagram for the protein surface characterization using an invariant descriptor. Protein structures in the figure are produced using PMV [3].

Figure 2 shows a schematic diagram of the general approach. The method has been applied on three protein families: *uracil-DNA glycosylase*, *estrogen receptor*, and *cell division protein kinase 2*. These are the same protein families used in a recently published work [8]. We also tested on *epidermal growth factor (EGF)* and *cyclooxygenase-2 (COX-2),* two protein families that are known to play a role in cancer. Below, we provide more details on the steps enumerated above.

### 3.1. Surface Generation

For a given protein, we first generate its Connolly surface [36] at a given atomic radii, using the MSMS program [37], based on which the dot surface is generated. This dot surface is stored in a vertex file. We have used a probe radius of 1.4 Å in all our experiments. Next, MATLAB Bioinformatics Toolbox (Mathworks Inc, Natick, Mass, USA) was used to extract the protein chains and to generate the residue coordinates in each chain. In this step, the chains are extracted while preserving the coordinates of the C_*α*_ atoms and their respective residue types by extracting the information from the PDB and the vertex files.

### 3.2. The Invariant Descriptor

#### 3.2.1. Surface Patches

To capture protein structure similarity and to avoid the computational complexity and the time-consuming problem of aligning 3D protein structures, we propose the use of a global rotational-invariant descriptor to represent overlapping patches on the protein surface. A patch is defined as a circular region with a specified radius, centered on the C_*α*_ position of a surface residue. For each residue on the surface (the central residue), we construct a surface patch by recording its residue type, and consider all residues within a certain distance threshold (*τ*_*p*_) as part of the patch (see Figure 2). Thus, the proposed surface descriptor is composed of 20 distinct descriptions, one for each protein residue type. For the local descriptor, this is constructed from only information from the patch. For the global descriptor, this is constructed by combining information from patches with the same central residue. 

The local invariant descriptor for the patch is created by calculating the distribution of distances between the central residue and all other surface residues within the patch. Additionally, the residue cooccurrences within the patch are also recorded as a part of the local descriptor. Each local descriptor is represented in a matrix **D**_**A**_ of size (20 + 1)×(*b* + 1), where the rows correspond to the 20 distinct protein residue types, plus an extra row to describe the summary distance distribution within the patch. The columns represent the individual bins used to capture the distance distributions (total of *b* bins), plus an extra column to represent the summary of the residue cooccurrences. To reduce the computational time and space requirement, unlike in [8] we define patches only for surface residue positions, rather than for each vertex on the dot surface (the number of vertexes is much more than the number of residues). Therefore, for a given chain, the number of local invariant descriptors will be equal to the number of surface residues. Yet, this number can vary from tens to hundreds and sometimes to thousands of surface residues. Using a huge number of local invariant descriptors for one chain to perform matching will be very time-consuming. To further reduce the computational requirements, for a given chain, we compute a global rotational-invariant descriptor by combining the 20 distinct residue-specific descriptors. For a given residue type, the global descriptor is constructed by taking the average of all patch descriptors with a given residue type as the central residue (see Figure 2). We consider three ways to represent and use the global surface descriptor, as explained below. 

#### 3.2.2. Distance Distribution (DD2)

The basic idea of using the distance distribution is that similar functional proteins should have a similar distribution of distances between the residues on their surfaces. The patch descriptor captures the distribution in two forms. The *first form* is a detailed distance distribution between the central residue in the surface patch and each of the other residues on the patch. To achieve this, a uniform distribution of the distances is assumed and the total number of bins *b* is used to estimate the probability distribution of finding a pair of residues at one of the *b* ranges. The *second form* is the global distance probability distribution. In this form we estimate the probability of observing any given residue within a patch in a particular distance range from the central residue. In this paper, we study the use of the global distance distribution in identifying similar protein surfaces, and possibly proteins with similar functions. Consequently, the question to be answered is, given a central residue of a specific type, what is the distance distribution for the residues around this central residue? That is, we seek Pr⁡{*d* | *R*_*c*_}, the probability of observing distance *d* between a central residue of type *R*_*c*_ and any other residue. We expect that the distance distribution should be similar for surface patches from functionally similar proteins.

#### 3.2.3. Residue Cooccurrences (RCs)

Given that surface structures are more conserved than sequence over evolution [15, 17], we expect that functionally similar proteins are likely to have similar surface residues, even though the order of such residues may have changed. This intuition is captured using residue cooccurrences on the protein surface. Using the distance distribution globally provides an idea of how the distances from the central residue are distributed in the protein surface patch. However, there is no constraint on, or indication of, which residues are involved in the formation of these distributions. The co-occurrence of a given residue with the central residue is calculated as the number of times the residue occurs on a patch with the same central residue. Thus, the main problem would be to find the probability of observing residue say, *R*_*i*_, given a central residue, say *R*_*c*_. Again, we expect the probability Pr⁡{*R*_*i*_ | *R*_*c*_}, to be similar for protein surfaces from functionally similar proteins. We note that the surface co-occurrence does not depend on the specific distance between the residues involved, as far as *R*_*i*_ is within the patch. 

#### 3.2.4. Distance-Residue Cooccurrences (DRCs)

The above have considered the distance and the co-occurrence separately. The DRC combines the general distance distribution (represented as a row vector, **sum** **C** in matrix **D**_**A**_) and the residue cooccurrences (represented as a column vector, **sum** **R** in matrix **D**_**A**_) in describing the protein surface (see Figure 2). The residue-distance co-occurrence vector is defined as follows: *D*_RC_ = (**sum** **C**°**sum** **R**^*T*^), where ° is the concatenation operator and **X**^*T*^ stands for the transpose of **X**. *D*_RC_ is used to compute the conditional probability Pr⁡{*d* | *R*_*c*_, *R*_*i*_}, that is, the probability of observing the distance *d* between residues *R*_*c*_ and *R*_*i*_ given that *R*_*c*_ is the central residue in the patch. We expect that the residue co-occurrence (**sum** **R**, or RC) should carry more distinctive functionally relevant information than the general distance distribution (**sum** **C**, or DD2), since surface residue cooccurrences are likely to be more conserved over evolution. By combining both vectors, we can account for both the geometry of the protein surface and the distribution of specific residues within specific distances on the surface. Using both vectors brings in some biological relevance in the analysis and is likely to lead to improved results in the identification of functionally similar protein surfaces.

### 3.3. Matching and Classification

Given two proteins, say Protein 1 and Protein 2 we characterize them using their global descriptors, say *D*_*g*1_ and *D*_*g*2_ respectively. In this work, the global descriptor could be the distance distribution (DD2), residue cooccurrences (RCs), or the distance-residue cooccurrences (DRCs).

**Distance Distribution**

For matching using the distance distribution we create a vector *D*_*g*1*d*_ that is composed of the 20 global distance distributions represented by all **sum** **C** vectors from each descriptor. *D*_*g*1*d*_ is defined as *D*_*g*1*d*_ = (*D*_*d*1_°  *D*_*d*2_°⋯°*D*_*d*20_), where *D*_*d*1_, *D*_*d*2_,…, *D*_*d*20_ are the distance distributions from each residue type on the surface of Protein 1. Repeat the same process for Protein 2 to create *D*_*g*2*d*_. Then we perform matching using the simple Euclidean distance: $D_{12}=\sqrt{\sum_{i=1}^{n}{\left[D_{g1d}\left(i\right)-D_{g2d}\left(i\right)\right]}^{2}}\;$.

**Residue Cooccurrences**

For Protein 1 we create a vector *D*_*g*1*c*_ that combines the 20 residues co-occurrence vectors (denoted **sum** **R**), defined as *D*_*g*1*c*_ = (*D*_*c*1_^*T*^°  *D*_*c*2_^*T*^°⋯°*D*_*c*20_^*T*^), where *D*_*c*1_^*T*^, *D*_*c*2_^*T*^  ,…, *D*_*c*20_^*T*^ represents **sum** **R**_1_^**T**^, **sum** **R**_2_^**T**^, and **sum** **R**_20_^**T**^. Similarly, we compute *D*_*g*2*c*_. Matching is performed using the Euclidean distance between *D*_*g*1*c*_ and *D*_*g*2*c*_.

**Distance-Residue Cooccurrences**

Here, we create a vector **DRC** that is comprised of all of the distance distributions as well as the residue cooccurrences. For Protein 1, we have DRC_1_ = (*D*_*d*1_°  *D*_*c*1_^*T*^° *D*_*d*2_°  *D*_*c*2_^*T*^°⋯°*D*_*d*1_°*D*_*c*1_^*T*^). Similarly we obtain DRC_2_ for Protein 2. Again for simplicity, matching is performed using the Euclidean distance. Clearly, other distance measures could be used.

**Classification**

Having computed the surface descriptors and the distance between protein surfaces using the descriptors, one may be interested in determining whether a given unknown protein belongs to some known protein family. Using some training data, we can compute surface descriptors for the known family, and based on these perform the required classification. Classification is performed using Weka [38, 39], an open-source software for machine leaning that provides a suite of classification algorithms.

## 4. Results and Discussion

### 4.1. Datasets and Environment

We performed experiments to test the performance of the proposed protein surface descriptor in two protein structure analysis tasks, namely, classifying proteins into their most likely functional groups, and ranking and retrieval of protein surfaces. We used two datasets for the experiments. DATASET-A contained information from three protein families: *uracil-DNA glycosylase*, *cell division protein kinase 2*, and *estrogen receptor*. This was created by scanning the PDB and selecting the protein structures with protein chains belonging to one of the three families. We were able to extract 416 chains that belong to 243 proteins in the PDB. The dataset is distributed as follows: 91 chains from 46 distinct proteins for *uracil-DNA glycosylase* (Group1), 186 chains from 95 distinct proteins for *estrogen receptor* family (Group2), and 139 chains from 102 distinct proteins from *cell division protein kinase 2* (Group3). We used DATASET-A basically to train the system, and perform initial testing. DATASET-B contained protein structures from two families, namely *cyclooxygenase-2 (COX-2*) (51 proteins, 95 chains) and *epidermal growth factor (EGF)* (67 proteins, 71 chains). We then extracted protein structures from the PDB that have 10 or less chains and ignored the rest. This resulted in a total of 15,386 protein chains form 6,261 unique proteins. DATASET-B included all structures in DATASET-A. We used DATASET-B for a more comprehensive scan of the PDB, in the quest for potentially novel structures that may be related to the two families. Experiments were performed using a SONY VAIO personal computer, with Intel Core 2 Duo Processor T8100, running at 2.10 GHz, with 2 GB of main memory. Programs were written using Matlab (Mathworks Inc, Natick, Mass, USA) with the Bioinformatics Toolbox. We set probe radius = 1.4 Å and patch distance threshold *τ*_*p*_ = 10 Å. For distance distributions, we used a fixed number of bins, *b* = 5. Classification was performed based on algorithms implemented in Weka [38, 39] version 3-6-4.

### 4.2. Classification Performance

**Figure 3:**
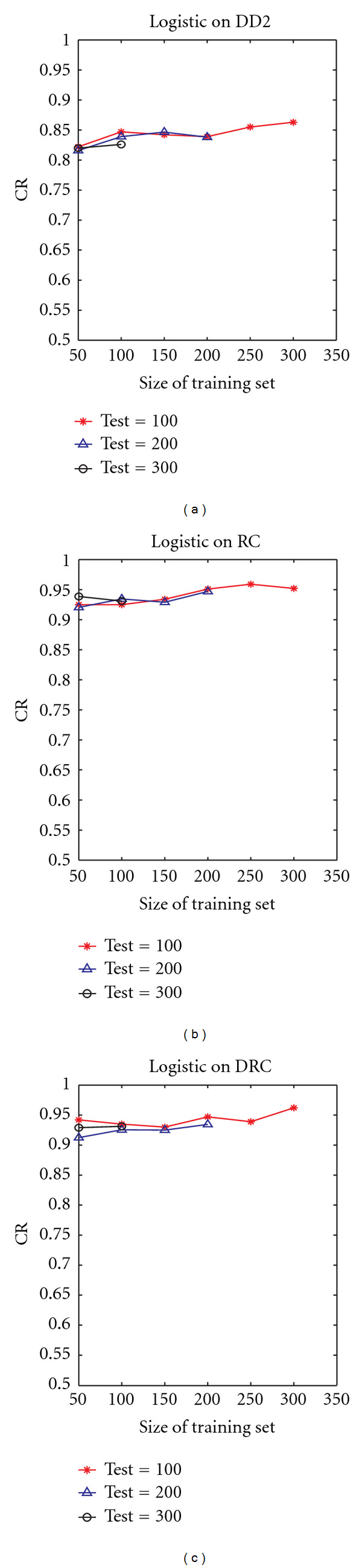
Variation of classification rate (CR) with size of training set using the proposed descriptors DD2 (a), RC (b), and DRC (c). Results are shown for the average over 10 runs, using logistic regression as the classifier.

We divide DATASET-A into training and testing sets and apply different classifiers on the different descriptors proposed. In all our experiments, the training sets were kept very separate from the testing sets, with no overlap between the two. Classification performance is measured in terms of classification rate based on the three protein families in the dataset. We tested the method using various classifiers implemented in Weka, such as Naïve Bayes, logistic regression, and simple logistic classifier. We report results mainly for the logistic regression. First, we explore the impact of the size of the testing set and of the training set on the classification performance using the proposed approach. We varied the size of the training set (from 50 to 300), while keeping the size of the testing set fixed. We then checked the performance using fixed testing sets of size 100, 200, and 300.  Figure 3 shows the results. 

The figure shows that applying the distance distribution (DD2) alone resulted in the lowest performance accuracy as compared to using the residue cooccurrences (RCs) or distance-residue cooccurrences (DRCs). Yet, our definition of the distance distribution shows encouraging results. A steady improvement in performance with increasing training set size can be observed when using DD2 alone, peaking at about 87% with a training size of 200 and testing size of 100. The distinctiveness of our approach is the use of residue cooccurrences on the protein surface. This approach assumes that functionally similar surface proteins have similar residue cooccurrences within a small local surface region.  Figure 3 (middle plot) shows that classification using residue cooccurrences (RCs) provided a significant improvement in the classification rate. A similar improvement was observed using other classifiers, such as Naïve Bayes. Using the RC descriptor, we can achieve an accuracy rate of 94% using a small training set (50 samples) and six times larger testing set (300 chains). This shows the robustness of the residue cooccurrences, even when using a few training samples. We observe that the performance using DD2 was not as robust (about 81% using small training set, peaking at about 87% using 200 training samples). 

**Figure 4:**
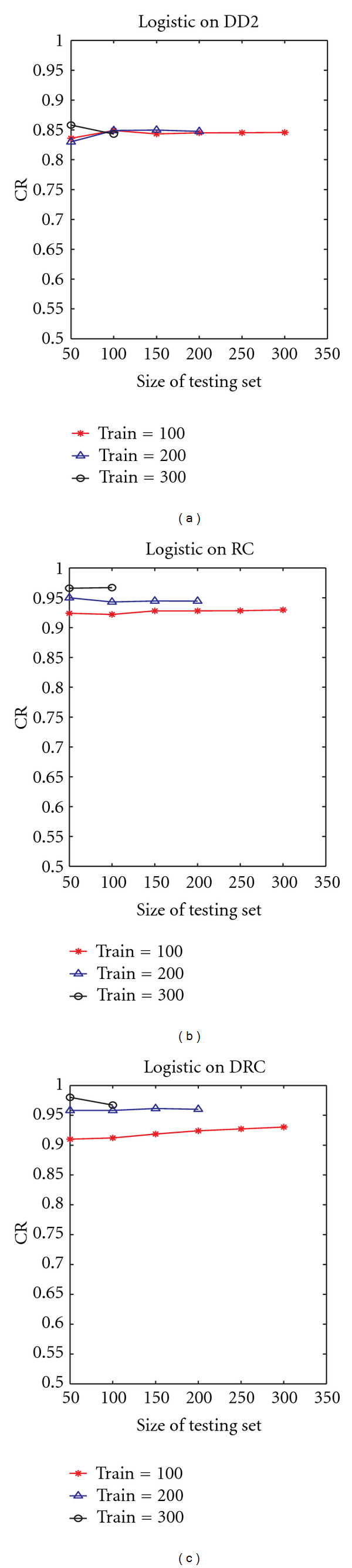
Variation of classification rate (CR) with size of testing set using the proposed descriptors DD2 (a), RC (b), and DRC (c). Results are shown for the average over 10 runs, using logistic regression as the classifier.

**Figure 5:**
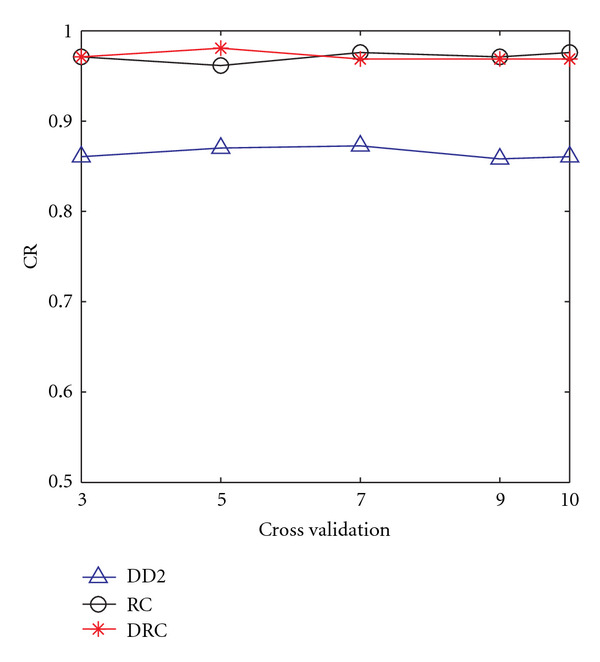
Summary classification performance using *n*-fold cross validation (the *x*-axis is for varying *n*).

The use of distance-residue co-occurrence presents a steadier improvement in the classification rate. Using the DRC raised the accuracy rate to 99% using the simple logistic classifier on a training set of 150 and testing set of 100 (data not shown). We can observe the significant difference between the results of DD2 (which did not use information on residue cooccurrences) and RC and/or DRC (both of which used residue cooccurrences).  Figure 4 shows a corresponding performance measurement with varying size of the testing set, while keeping the training set size fixed. As expected, there is a general slight decrease in performance with increasing size of the test set. The case of DRC using a training set size of 100 seemed to increase slightly with increasing testing set size. The increase is however within a small range (from 0.91 to 0.93). This shows a steady performance over increasing size of the testing set. Overall trends are similar to Figure 3, with RC and DRC performing much better than DD2. Similar trends were also observed using other classification algorithms. The overall classification performance is summarized in Figure 5, which shows the results of the three proposed schemes using *n*-fold cross validation, for different values of *n*. 

### 4.3. Ranking and Retrieval

In this section, we explore the effectiveness of our approach on the problem of search and retrieval of protein surfaces. Given a query protein, we study whether our approach has the robustness to place most of the functionally similar proteins in the top hits of the retrieved surfaces. Here, a query protein from each of the three groups is used to screen the entire DATASET-A (416 samples) and provide a ranking based on the similarity. Thus, each protein structure is ranked against the query, (from 1 to 416), where a lower rank (smaller distance) implies more similarity to the query. After that, we search over the retrieved proteins to find which ranks the functionally similar proteins (i.e., proteins in the same functional group) have attained.  Table 1 shows the ranking produced using the proposed descriptor, for three query samples, one for each group. Results are shown only for DRC. RC produced a slightly better ranking (especially for *uracil-DNA glycosylase* family (Group 1)), while DD2 was worse than both RC and DRC). Overall, for Group 2 and Group 3, the Top 30 ranked proteins belonged to the corresponding family, while Group 1 was more difficult. 

**Figure 6:**
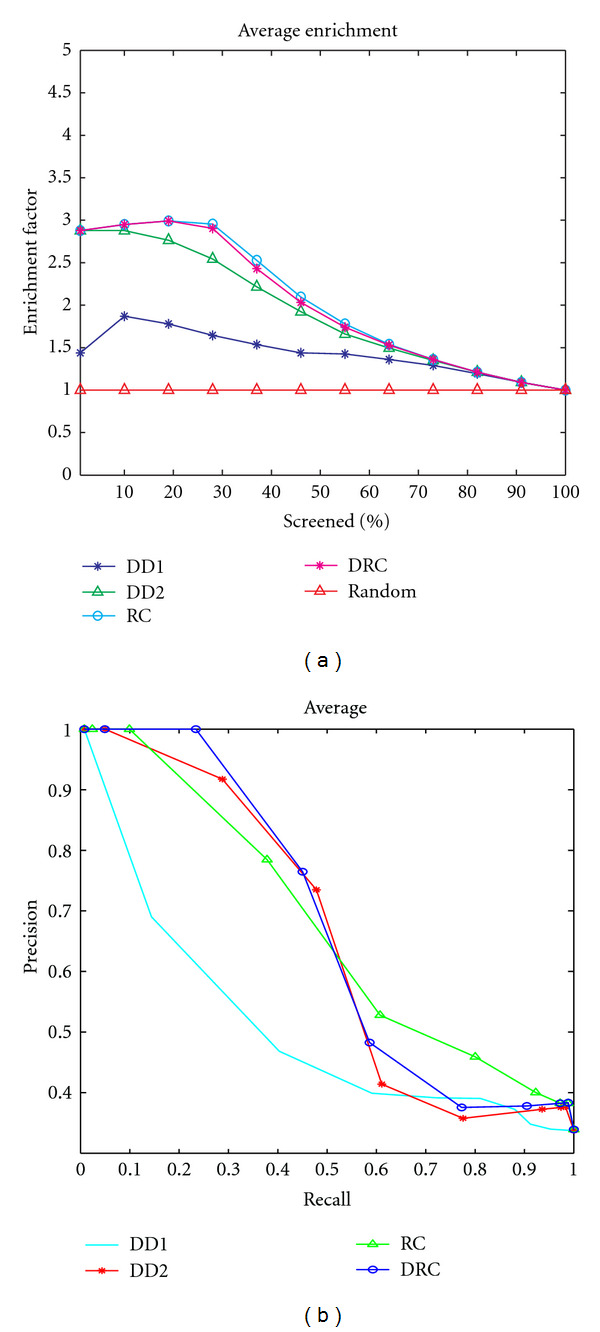
Ranking and retrieval performance for the proposed methods. (a) Enrichment plot for screening protein structures using the proposed descriptors. Results are average for 5 query proteins from *cell division protein kinase 2* family (Group 3), using DATASET-A (416 protein chains). (b) Average precision and recall for three queries, one for each group in DATASET-A. DD1 corresponds to the distance distribution proposed in [11], as described in Section 2 (see Section 4.5 on comparison with related methods).

We further measured the performance of our approach using the enrichment plot. The enrichment plot essentially measures how well a given ranking or retrieval system performs, when compared with a random selection of the data samples. At a given percentage of database screening, the enrichment factor is computed as the ratio *N*_obs_/*N*_exp⁡_, where *N*_obs_ = number of functionally similar proteins observed or retrieved by the system, and *N*_exp⁡_ = number of functionally similar proteins expected by random selection. For an effective system, we expect that most of the functionally similar proteins should be observed after a small percentage of screening. That is, the top hits should contain mainly functionally similar proteins, and hence the enrichment factor should be high after a small percentage probe of the database, and gradually decrease towards 1 (which corresponds to random selection).  Figure 6(a) shows a plot of the average enrichment factor using 5 queries from Group 3. The enrichment plot shows that our proposed method provides better results as we screen a small percentage of the dataset. In most of the cases, our method retrieved about three times better than the expected random retrieval in the first 10% of screened proteins. As we increase the percent of screening, the retrieval degrades, since we are more likely to have retrieved most, if not all of the similar proteins after a small percentage of the screening. Thus, subsequent retrievals will lead to spurious results.

### 4.4. Screening Protein Surfaces in PDB

Encouraged by the results in classification and ranking using the proposed descriptors, we now performed a larger scale experiment, by screening the entire protein structures in PDB, using the protein chains in DATASET-B, with members of the *COX-2* and *EGF* families as the query. The main objective was to see how the proposed descriptors will perform on a large scale, and to see if the methods could predict potentially novel functional linkages between any of the families and other proteins in PDB. For this task, we used only PDB files with 10 or less chains, and ignored the rest. This resulted in a total of 15,386 protein chains from 6,261 unique proteins.  Table 2(a) shows the ranking results produced by screening the PDB files based on the proposed descriptors, using a member of the *EGF* family as a query.  Table 2(b) shows corresponding results using a member of *COX-2* family. Results are shown only for the DRC descriptor. Generally, similar results were obtained using RC. We can notice that some of the unknown proteins (annotated as “uncharacterized”) were placed in the Top-50 rankings, implying a possible relationship with the respective families. 

### 4.5. Comparison with Related Methods

The use of distance distributions for protein surface analysis was studied by Binkowski et al. [11]. As earlier discussed, they did not consider the specific residues in constructing the distributions. Their distance distribution (labeled as DD1 in this work) is obtained by removing the reference to the specific residue at the center of the patch (see Figure 2). Our use of surface residue cooccurrences and combining these with the residue-specific distance distributions are novel methods introduced in this paper. Tables 3(a) and 3(b) compare the overall classification performance using DD1 with those obtained with the proposed descriptors. 

Figure 6 also shows the comparative performance using both the enrichment plots, and precision and recall. We define precision and recall at a given distance threshold as follows: precision = (number of correct retrievals at the threshold)/(number of total retrievals at the threshold). Recall = (number of correct retrievals at the threshold)/(number of total true matches expected at the threshold). Here, using the ranked results, for a given query and a given rank, the number of expected true matches will be **min**{rank, query group size}. This is similar to the definition used in [28]. We performed queries on DATASET-A using query proteins from each of the three groups and computed the average precision and recall for each descriptor. We then computed the area under the curve (AUC) for the average precision-recall plots. The results were as follows: DD1 (0.501052), DD2 (0.649412), RC (0.668303), and DRC (0.66759). Although the databases used are different, these results compare well with the results reported by Sael and Kihara [28], where they evaluated the retrieval performance of four surface characterization methods, based on the Zernike representation. The maximum AUC reported using standard resolution surfaces was 0.608 (without length filtering) and 0.628 (with length filtering). Yin et al. [8] proposed a fingerprint-based method, using surface alignment on selected surface patches. Their method constructs an initial patch on every vertex on the dot surface, and requires computation of geodesic distances on the surface, two very time-consuming processes. Our method neither requires surface alignment, nor expensive computations on the surface, beyond the surface generation process. Patches are generated only on positions of the surface residues, rather than over all the vertices on the generated protein surface.

### 4.6. Computation Time

The most time consuming part was for preprocessing, as needed to construct the protein surfaces and extract the protein chains. The construction of the protein surface from the original PDB files required about 4.065 seconds, running on *Cygwin* (a version of Linux for Microsoft Windows). Extraction of the protein chains and the C_*α*_ atoms was performed using Matlab Bioinformatics Toolbox (Mathworks Inc., Natick, Mass, USA), and required 32 seconds per PDB file. Construction of the descriptors after the above steps took an average of 0.7 seconds per PDB file. Querying DATASET-B (15,386 chains, 6,261 unique PDB files) using the DRC descriptor required an average time of 0.28 seconds for each query PDB file.

## 5. Conclusion

We have introduced a novel approach to the description and characterization of protein surfaces. The proposed approach captures the surface structure of the protein by utilizing local patches defined only on the positions of surface residues, rather than over all surface vertices, or over all the surface atoms. We make residue cooccurrences on the surface a central part of the descriptor. The novelty of this approach can be observed by the ease of handling both local and global variation on the surface (using local and global descriptors). Moving from local to global not only reduces the computational problem of matching 3D structures, but also facilitates direct comparison between protein structures of different sizes. By avoiding the construction of the complete 3D surface and retaining only the surface C_*α*_ to do the analysis, the need for surface alignment of the 3D structure is eliminated. Further, we do not need to perform any geometrical transformation to insure reliable matching. This is very important for rapid analysis over a large database, such as the PDB. 

We showed results on the performance of the proposed methods in functional classification of proteins into their putative families, based on the surface information. We further compared the results using enrichment plots, and the standard measures of precision and recall. For the three protein families used, we obtained an area under the curve for precision and recall of 0.6494 (DD2), 0.6683 (RC), and 0.6676 (DRC). A screening of the PDB using *COX-2* and *EGF* family members showed that the proposed methods ranked related family members in the Top-20 hits, with a number of uncharacterized proteins also retrieved. It will be interesting to perform further biological lab experiments to verify if any of the retrieved uncharacterized proteins are truly related to the respective families to which they share similar surfaces (as determined by our surface descriptors).

## Figures and Tables

**Table 1a:** (a) DRC on query protein 1UDI chain I (Group 1)

Protein PDB ID	Chain	Rank	Distance
1UDI	I	1	0
2ZHX	B	2	2.1306
1LQM	B	3	2.3509
1LQG	C	4	2.4589
2UUG	C	5	2.5353
1EUI	C	7	2.5920
2ZHX	L	8	2.6104
1UGH	I	10	2.6349
2UGI	A	15	2.6809
1UGI	E	16	2.6872
2ZHX	H	19	2.6969
2ZHX	D	21	2.7006
2ZHX	N	22	2.7017
2ZHX	J	23	2.7141
1UGI	G	25	2.7261
1EMJ	A	42	2.7758
2BOO	A	45	2.7808
1UGI	D	47	2.7868
2OWR	B	50	2.7952
2J8X	D	61	2.8129
1LQG	D	70	2.8263
1Q3F	A	90	2.8533
2UUG	D	99	2.8675
1UGI	A	101	2.8689
2OWR	C	110	2.8749
2ZHX	F	116	2.885
2OWQ	B	129	2.8977
1SSP	E	141	2.9115
1UGI	C	142	2.9117
2ZHX	A	147	2.915

**Table 1b:** (b) DRC on query protein 1QKN chain A (Group 2)

Protein PDB ID	Chain	Rank	Distance
1QKN	A	1	0
2J7X	A	2	1.3769
2J7Y	A	3	1.5753
1QKM	A	4	1.6793
1NDE	A	5	1.7368
2GIU	A	6	1.7371
1L2I	A	7	1.7460
1U3R	B	8	1.7670
3ERD	A	9	1.7683
3OS9	A	10	1.7715
2IOG	A	11	1.7738
3LTX	C	12	1.7742
1YIM	A	13	1.7854
3ERT	A	14	1.7966
1U3Q	D	15	1.8009
1YY4	A	16	1.8090
2OUZ	A	17	1.8126
1YIN	A	18	1.8152
1XP6	A	19	1.8260
2AYR	A	20	1.8269
3OS8	D	21	1.8311
2QH6	A	22	1.8312
3OSA	A	23	1.8367
1L2J	A	24	1.8385
2JJ3	A	25	1.8438
1G50	A	26	1.8490
3OS8	A	27	1.8509
2FSZ	A	28	1.8518
2QGW	A	29	1.8604
1UOM	A	30	1.8683

**Table 1c:** (c) DRC on query protein 1YKR chain A (Group 3)

Protein PDB ID	Chain	Rank	Distance
1YKR	A	1	0
2UZO	A	2	1.0396
2R3O	A	3	1.0810
3PXY	A	4	1.0846
3PY1	A	5	1.0940
2WMA	A	6	1.1389
2IW6	A	7	1.1609
3NS9	A	8	1.2043
3IGG	A	9	1.2141
2WFY	A	10	1.2179
2C5Y	A	11	1.2270
3DDP	A	12	1.2280
2J9M	A	13	1.2284
2R3J	A	14	1.2374
2R3L	A	15	1.2402
3PXR	A	16	1.2422
2DUV	A	17	1.2534
1W8C	A	18	1.2586
3DOG	A	19	1.2793
2V22	A	20	1.2822
2R3P	A	21	1.2883
2V22	C	22	1.2963
3IG7	A	23	1.3207
2JGZ	A	24	1.3275
2R64	A	25	1.3303
2WHB	A	26	1.3381
2VTN	A	27	1.3458
3LFN	A	28	1.3476
2WIP	A	29	1.3514
2BKZ	A	30	1.3580

**Table 2a:** (a) Top 50 hits using DRC for a query protein structure from the EGF family on DATASET-B. Annotations in bold correspond to members of the EGF family, predicted proteins, or uncharacterized proteins

Protein	Chain	Distance	Protein name annotation	Rank
2a2q	L	0.0000	**COAGULATION FACTOR VII**	1
2fir	L	1.4925	**COAGULATION FACTOR VII LIGHT CHAIN**	2
2zp0	L	1.5545	**FACTOR VII LIGHT CHAIN**	3
1wtg	L	1.5628	**COAGULATION FACTOR VII**	4
1wun	L	1.5844	**COAGULATION FACTOR VII**	5
2b8o	L	1.6305	**COAGULATION FACTOR VII LIGHT CHAIN**	6
2zwl	L	1.6379	**FACTOR VII LIGHT CHAIN**	7
2zzu	L	1.6536	**FACTOR VII LIGHT CHAIN**	8
1wqv	L	1.6816	**COAGULATION FACTOR VII**	9
2ec9	L	1.6832	**COAGULATION FACTOR VII**	10
1dan	L	1.7655	**BLOOD COAGULATION FACTOR VIIA**	11
1wss	L	1.7659	**COAGULATION FACTOR VII**	12
2puq	L	1.7692	**COAGULATION FACTOR VII**	13
1fak	L	1.7934	**PROTEIN (BLOOD COAGULATION FACTOR VIIA)**	14
2b7d	L	1.8024	**COAGULATION FACTOR VII**	15
6acn	A	1.8061	ACONITASE	16
2aer	L	1.8120	**COAGULATION FACTOR VII**	17
2aei	L	1.8164	**COAGULATION FACTOR VII**	18
2flr	L	1.8196	**COAGULATION FACTOR VII**	19
3ela	L	1.8668	**COAGULATION FACTOR VII LIGHT CHAIN**	20
1z6j	L	1.8859	**COAGULATION FACTOR VII**	21
2f9b	L	1.9027	**COAGULATION FACTOR VII**	22
3phs	A	1.9169	CELL WALL SURFACE ANCHOR FAMILY PROTEIN	23
3n54	B	1.9263	SPORE GERMINATION PROTEIN B3	24
3qbp	B	1.9264	FUMARASE FUM	25
3ma9	L	1.9367	TRANSMEMBRANE GLYCOPROTEIN	26
3mt0	A	1.9921	**UNCHARACTERIZED PROTEIN PA1789**	27
3lgu	A	2.0004	PROTEASE DEGS	28
3m7i	A	2.0071	TRANSKETOLASE	29
1qfk	L	2.0096	**PROTEIN (COAGULATION FACTOR VIIA (LIGHT CHAIN))**	30
3n9t	A	2.0169	PNPC	31
3pxz	A	2.0235	CELL DIVISION PROTEIN KINASE 2	32
2flb	L	2.0257	**COAGULATION FACTOR VII**	33
3lh1	A	2.0289	PROTEASE DEGS	34
3nlc	A	2.0300	**UNCHARACTERIZED PROTEIN VP0956**	35
3no5	C	2.0302	**UNCHARACTERIZED PROTEIN**	36
3msq	C	2.0347	PUTATIVE UBIQUINONE BIOSYNTHESIS PROTEIN	37
3ryk	A	2.0389	DTDP-4-DEHYDRORHSMNOSE 3,5-EPIMERASE	38
3m4a	A	2.0444	PUTATIVE TYPE I TOPOISOMERASE	39
3n3n	B	2.0444	CATALASE-PEROXIDASE	40
2R3G	A	2.0456	CELL DIVISION PROTEIN KINASE 2	41
2R3I	A	2.0465	CELL DIVISION PROTEIN KINASE 2	42
3o0r	L	2.0473	ANTIBODY FAB FRAGMENT LIGHT CHAIN	43
3n3p	B	2.0490	CATALASE-PEROXIDASE	44
3nfh	A	2.0492	DNA-DIRECTED RNA POLYMERASE I SUBUNIT RPA49	45
3qfk	A	2.0501	**UNCHARACTERIZED PROTEIN**	46
3n5h	F	2.0517	FARNESYL PYROPHOSPHATE SYNTHASE	47
3n3o	B	2.0529	CATALASE-PEROXIDASE	48
3o78	B	2.0548	CHIMERA PROTEIN OF PEPTIDE OF MYOSIN LIGHT CHAIN SMOOTH MUSCLE, GREEN FLUORESCENT PROTEIN, GREEN FLUORESCENT CALMODULIN	49
3luy	A	2.0570	PROBABLE CHORISMATE MUTASE	50

**Table 2b:** (b) Top 50 hits using DRC for a query protein structure from the COX-2 family on Dataset-B. Annotations in bold correspond to members of the COX-2 family, predicted proteins, or uncharacterized proteins

Protein	Chain	Distance	Protein name annotation	Rank
2zxw	B	0.0000	**CYTOCHROME C OXIDASE SUBUNIT 2**	1
2eil	B	1.3914	**CYTOCHROME C OXIDASE SUBUNIT 2**	2
2eij	B	1.5853	**CYTOCHROME C OXIDASE SUBUNIT 2**	3
3ag4	B	1.6147	**CYTOCHROME C OXIDASE SUBUNIT 2**	4
2dys	B	1.6991	**CYTOCHROME C OXIDASE SUBUNIT 2**	5
3ag1	B	1.8159	**CYTOCHROME C OXIDASE SUBUNIT 2**	6
2eim	B	1.8824	**CYTOCHROME C OXIDASE SUBUNIT 2**	7
3ag2	B	2.0173	**CYTOCHROME C OXIDASE SUBUNIT 2**	8
2occ	B	2.0631	**CYTOCHROME C OXIDASE**	9
3abl	B	2.1404	**CYTOCHROME C OXIDASE SUBUNIT 2**	10
2eik	B	2.1413	**CYTOCHROME C OXIDASE SUBUNIT 2**	11
3abm	B	2.1454	**CYTOCHROME C OXIDASE SUBUNIT 2**	12
1v55	B	2.1489	**CYTOCHROME C OXIDASE POLYPEPTIDE II**	13
2dyr	B	2.2044	**CYTOCHROME C OXIDASE SUBUNIT 2**	14
2ein	B	2.2628	**CYTOCHROME C OXIDASE SUBUNIT 2**	15
1v54	B	2.2976	**CYTOCHROME C OXIDASE POLYPEPTIDE II**	16
3n56	B	2.4226	INSULIN-DEGRADING ENZYME	17
3abk	B	2.4502	**CYTOCHROME C OXIDASE SUBUNIT 2**	18
3p42	A	2.4785	**PREDICTED PROTEIN**	19
3r2u	B	2.4846	METALLO-BETA-LACTAMASE FAMILY PROTEIN	20
3msu	B	2.4920	CITRATE SYNTHASE	21
3ag3	B	2.4950	**CYTOCHROME C OXIDASE SUBUNIT 2**	22
3ntd	B	2.5052	FAD-DEPENDENT PYRIDINE NUCLEOTIDE-DISULPHIDE OXIDOREDUCTASE	23
3ngi	A	2.5055	DNA POLYMERASE	24
7xim	B	2.5198	D-XYLOSE ISOMERASE	25
3mjy	A	2.5206	DIHYDROOROTATE DEHYDROGENASE	26
3nva	B	2.5391	CTP SYNTHASE	27
3lm3	A	2.5433	**UNCHARACTERIZED PROTEIN**	28
3ppn	B	2.5511	GLYCINE BETAINE/CARNITINE/CHOLINE-BINDING PROTEIN	29
3o98	B	2.5557	BIFUNCTIONAL GLUTATHIONYLSPERMIDINE SYNTHETASE/AM	30
3pom	B	2.5558	RETINOBLASTOMA-ASSOCIATED PROTEIN	31
3nt6	B	2.5656	FAD-DEPENDENT PYRIDINE NUCLEOTIDE-DISULPHIDE OXIDOREDUCTASE	32
5lym	B	2.5690	LYSOZYME	33
3n1y	B	2.5728	TOLUENE O-XYLENE MONOOXYGENASE COMPONENT	34
1occ	B	2.5772	**CYTOCHROME C OXIDASE**	35
3lxt	D	2.5860	GLUTATHIONE S TRANSFERASE	36
2q70	B	2.5922	ESTROGEN RECEPTOR	37
3l49	B	2.5930	ABC SUGAR (RIBOSE) TRANSPORTER, PERIPLASMIC SUBSTRATE-BINDING SUBUNIT	38
3pvq	A	2.5944	DIPEPTIDYL-PEPTIDASE VI	39
3puf	B	2.6032	RIBONUCLEASE H2 SUBUNIT B	40
3mve	B	2.6077	UPF0255 PROTEIN VV1_0328	41
3ld2	B	2.6143	PUTATIVE ACETYLTRANSFERASE	42
3ne6	A	2.6160	DNA POLYMERASE	43
3qae	A	2.6179	3-HYDROXY-3-METHYLGLUTARYL-COENZYME A REDUCTASE	44
3qh8	A	2.6197	BETA-LACTAMASE-LIKE	45
3m3r	A	2.6215	ALPHA-HEMOLYSIN	46
3nrb	B	2.6234	FORMYLTETRAHYDROFOLATE DEFORMYLASE	47
3n05	B	2.6240	NH(3)-DEPENDENT NAD(+) SYNTHETASE	48
3m2l	A	2.6324	ALPHA-HEMOLYSIN	49
3pns	B	2.6357	URIDINE PHOSPHORYLASE	50

**Table 3a:** (a) Overall classification rate using different classifiers (300 training samples, 100 testing samples from DATASET-A)

Classifier	Descriptor			
	DD1	DD2	RC	DRC

Naïve bayes	58%	86%	94%	91%
Logistic	58%	85%	99%	97%
Simple logistic	58%	89%	98%	91%

**Table 3b:** (b) Overall classification rate using different classifiers (100 training samples, 300 testing samples from DATASET-A)

Classifier	Descriptor			
	DD1	DD2	RC	DRC

Naïve bayes	55%	74%	94%	93%
Logistic	62%	88%	89%	94%
Simple logistic	63%	85%	91%	90%
